# Multi‐Scale Habitat Drivers of Hainan Gibbon Distribution: Integrating Macro‐Environmental and Fine‐Scale Ecological Factors for Targeted Conservation of Threatened Species

**DOI:** 10.1002/ece3.74519

**Published:** 2026-10-26

**Authors:** Shuai Liu, Dexu Zhang, Chenglin Cai, Yidong Zhou, Kening Lu, Yuan Chen, Wenxing Long, Eric I. Ameca

**Affiliations:** ^1^ School of Ecology ‐ Hainan University Haikou China; ^2^ The Research and Conservation Centre of Hainan Gibbon Hainan University Haikou China; ^3^ The National Research and Conservation Centre of Gibbon Bawangling China; ^4^ School of Ecology and Environment North China University of Water Resources and Electric Power Zhengzhou China; ^5^ School of Tropical Agriculture and Forestry ‐ Hainan University Haikou China; ^6^ IUCN SSC ‐ Primate Specialist Group Gland Switzerland

**Keywords:** activity patterns, conservation management, habitat characteristics, *Nomascus hainanus*, population distribution

## Abstract

As habitat fragmentation intensifies due to Anthropocene impacts, ecological variation across spatial scales may cause conservation strategies to misalign with species' actual needs. Understanding how cross‐scale spatial heterogeneity in ecological variables shapes species distributions is therefore critical to optimize conservation prioritization. The Critically Endangered Hainan gibbon is restricted to highly degraded forests, yet the interplay of ecological drivers shaping its current distribution, from macro‐environmental constraints to fine‐scale habitat selection, remains poorly quantified. This study integrates two‐year monitoring data from all seven known Hainan gibbon groups (GA‐GG, representing the global population) using a multi‐scale sampling design. We employed 395 population‐level and 135 group‐level plots to evaluate 23 ecological variables spanning macro‐ (e.g., climate) to micro‐scales (e.g., food plant diversity), identifying drivers of gibbon distribution. We found that population‐scale distribution is driven by climatic and topographic factors (e.g., precipitation, elevation), whereas group‐scale distribution responded to fine‐scale habitat features (e.g., food plant diversity, terrain ruggedness, crown area). GC distribution reflects optimal foraging (preference for food plant diversity), whereas GE adopts an energy‐conserving strategy (selecting complex terrain with high food plant abundance). Habitat suitability followed a J‐shaped threshold response at the population scale, and divergent responses at the group level (rapid in GE, stable in GC). These findings underscore the need for scale‐ and habitat‐specific conservation: expanding climatically and vegetatively suitable areas at the population level, restoring food availability in GE habitats, and maintaining stability in GC habitats. Our multi‐scale mechanistic framework provides actionable conservation insights, with applications for other range‐restricted species in fragmented landscapes.

## Graphical abstract

We revealed strong scale‐dependent divergence in the ecological drivers shaping Hainan gibbon distribution. Population‐level patterns were primarily constrained by macro‐environmental factors, whereas group‐level activity differed markedly between family groups in response to fine‐scale habitat features. By integrating these drivers into an ecological weighted index, we propose habitat restoration strategies tailored to both spatial scale and group‐specific ecological constraints.

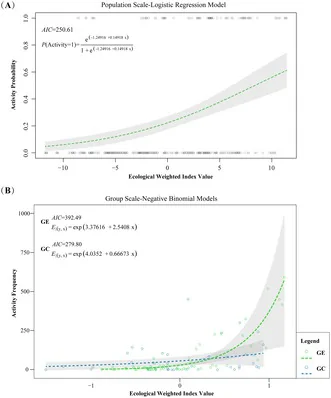

## 1 Introduction

In the Anthropocene era, the impacts of human activities and climate change on species and their habitats remain escalating and urge targeted conservation efforts to prevent extinctions (Walther et al. 2002; IPBES 2024). The success of these efforts, however, depends on a nuanced understanding of the distribution patterns of species as well as their habitat requirements across different spatial scales (Stein et al. 2014). At the landscape level, habitat protection and management (e.g., large‐scale reforestation and corridors connecting different habitat types, invasive species management) can benefit entire populations through the establishment of protected areas, law enforcement, and enhancements of degraded habitat (UN 2015; Liang et al. 2018; Kearney et al. 2023; Timms and Holden 2024). Below the landscape level, localized protection and habitat management that focuses on subpopulations or individual family groups can improve food availability and shelter (Estrada et al. 2017). In addition, long‐term monitoring of threats along with tailored conservation planning (e.g., microhabitat interventions based on foraging flexibility) can improve habitat suitability, support stable group sizes and structures, and enhance resilience to environmental changes (Behie et al. 2019). Given the interdependence of ecological processes across scales, species conservation efforts can benefit from cross‐scale approaches that align local interventions with the species' needs across the broader landscape, thereby minimizing the risk of implementing interventions that could lead species to ecological traps (Battin 2004).

At the landscape level, studies on non‐human primates have demonstrated that temperature and precipitation significantly influence both habitat suitability and species range (Lehmann et al. 2010; Zhao et al. 2023). For example, African apes (*Pan* spp. and *Gorilla* spp.) are projected to face substantial range reductions under future climate scenarios, with rising temperatures (+5.2°C) and increased rainfall (+15%) reducing suitable habitat by ~75% for gorillas and 10% for chimpanzees (Lehmann et al. 2010). Likewise, gibbons (e.g., *Nomascus concolor* , *N. hainanus* , *Hoolock tianxing* ) have already experienced a 71% range contraction between 1951 and 2020 driven by both climate (temperature and precipitation) shifts and human‐induced deforestation (Zhao et al. 2023), while howler monkeys (*Alouatta* spp.) and spider monkeys (*Ateles* spp.) in the Neotropics have experienced climate‐related range shifts associated with seasonal rainfall and fruiting variability (González‐Zamora et al. 2011). Vegetation characteristics are also important for primate distribution. In a study assessing future distributions of 26 primate species in China, habitat suitability models incorporating ecological variables such as NDVI and the presence of protected areas (PAs) revealed that higher NDVI values—indicative of denser vegetation—consistently enhanced habitat suitability and species presence probabilities (Li et al. 2024). Moreover, land use and forest type are crucial for shaping species dispersal and maintaining population by influencing habitat connectivity. For instance, Asian primates such as the proboscis monkey ( *Nasalis larvatus* ) and Tonkin snub‐nosed monkey ( *Rhinopithecus avunculus* ) are particularly vulnerable to habitat fragmentation, as land use change reduces habitat connectivity, amplify edge effects and isolation, and lead to population declines (Fahrig 2003; Haddad et al. 2015; Boonratana 2019). These macro‐level variables—including climate (e.g., temperature and precipitation), vegetation characteristics (e.g., NDVI), land use and forest type—are particularly crucial for species with narrow habitat and diet breadth, as environmental changes can lead to reduced food and shelter availability (Ameca et al. 2024), increased predation risk (Bettridge and Dunbar 2012), increased exposure to pathogens (Patouillat et al. 2024), and disruptions of social organization (Pavelka et al. 2003). However, the importance of these variables may vary with spatial scale, and habitat quality may be equally or more influential at smaller scales (i.e., group scale; Fan et al. 2011). Thus, effective conservation strategies must integrate multi‐scale assessments, addressing landscape‐level connectivity while ensuring habitat quality.

Fine‐scale environmental variables such as canopy structure, plant diversity, and resource distribution play a crucial role in shaping primate activity patterns, time budgets, and behavioral strategies (Chapman et al. 2018; Zhong et al. 2023). For instance, studies on lemur species (*Lemur* spp.) have shown that groups preferentially select areas with high canopy connectivity and larger mean tree diameters, where activity frequency is significantly higher (Chen et al. 2021). In addition, the diversity of food resources directly influences primate behavioral strategies (Clink et al. 2017). In the montane forests of the Wuliang Mountains in northern Yunnan, the western black crested gibbon ( *N. concolor* ) adopts a time‐minimizing strategy when food is abundant and switches to an energy‐conserving strategy during food scarcity, which enhances its survival and reproductive success (Ning et al. 2019). Furthermore, the availability of key food plants within the habitat can strongly influence primate foraging behavior. This pattern is evident in the western hoolock gibbon ( *Hoolock hoolock* ) inhabiting forest fragments in northeastern India, where fig species account for approximately 36% of the diet (Borah et al. 2018). These findings underscore how fine‐scale habitat features shape primate behavioral ecology, influencing both activity patterns and foraging strategies (Chan et al. 2020). In addition, the heterogeneity of habitat quality in fragmented landscapes can influence the adaptability of different groups and their preferences for specific ecological variables (Ni et al. 2014). Understanding these fine‐scale dynamics is essential for optimizing habitat restoration efforts and ensuring that conservation interventions align with species' behavioral ecology.

The Hainan gibbon ( *Nomascus hainanus* (Thomas, 1892)), one of the world's most endangered primates, has experienced a population decline of at least 80% over the past 45 years (Geissmann and Bleisch 2020), primarily due to hunting and habitat loss (Mootnick et al. 2012, Turvey et al. 2015). Currently, seven social groups (code‐named GA–GG), comprising 42 individuals, persist within 23.25 km^2^ of tropical forest in the Bawangling Branch of the National Park of Hainan Tropical Rainforest (NPHTR), China, underscoring the species' precarious survival status (Turvey et al. 2018). Despite substantial conservation efforts, the optimal strategy for its recovery remains contested. Some researchers advocate for habitat expansion to facilitate population growth and range extension (Zhang et al. 2022), while others emphasize habitat quality enhancement to improve survival and reproductive success (Chan et al. 2020). This debate likely arises from different scale‐dependent conservation priorities: at the landscape level, expanding suitable habitat is crucial for long‐term population viability (Zhang et al. 2025), whereas at the local level habitat quality improvements determine daily individual survival and reproductive outcomes (Liu, Zhang, Ameca, et al. 2025; Li et al. 2026). Furthermore, recent studies indicate that Hainan gibbons are increasingly using lowland secondary forests, suggesting ongoing habitat range shifts (Liu, Zhang, Zhang, et al. 2025). Differences in habitat preferences between family groups residing in relatively mature forests (GA–GD groups) and those in secondary forests (GE–GG groups) may reflect distinct ecological requirements (Zhang et al. 2022, 2024) but a comprehensive assessment of the factors driving these differences has not been carried out in any systematic way.

To address this knowledge gap, we examined how multiple ecological predictors influence the distribution of Hainan gibbons across both population and group scales. The selected predictors represent key ecological dimensions previously shown to influence primate distribution and habitat suitability, including climatic conditions, vegetation productivity, habitat structure, and food resource availability (Fan and Jiang 2008; Chapman et al. 2018). Focusing on the seven extant groups (GA–GG), we hypothesize that temperature, precipitation, and NDVI drive Hainan gibbon distribution at the population scale, whereas food availability and canopy structure primarily determine activity patterns at the group scale. In addition, we focused on groups C (GC) and E (GE) to test whether they exhibit distinct ecological preferences and activity strategies from each other given their divergent habitat characteristics (GC inhabits relatively mature forests, whereas GE inhabits secondary forests regenerated from historical pine plantations; Liu, Zhang, Ameca, et al. 2025).

By examining drivers across population and group scales in the critically endangered Hainan gibbon, this study provides valuable insights to guide targeted conservation strategies that align with the species' ecological needs at each spatial scale. Our approach offers a transferable model for other endangered species in the face of similar multi‐scale habitat drivers under rapid environmental change.

## 2 Materials and Methods

### 2.1 Study Area

Our study area is located within the Bawangling branch (18°48′–19°12′N, 108°55′–109°17′ E) of the NPHTR, Hainan Island, China (Figure 1). The entire known population of the Hainan gibbon, comprising seven family groups (GA–GG) along with several solitary individuals, resides in this area. The region exhibits distinct seasonal patterns, with a wet season from May to October and a dry season from November to April (Fan et al. 2021). As elevation increases, the primary vegetation transitions sequentially through tropical lowland rainforest, tropical montane rainforest, and tropical cloud rainforest (Jiang 2015).

**FIGURE 1:**
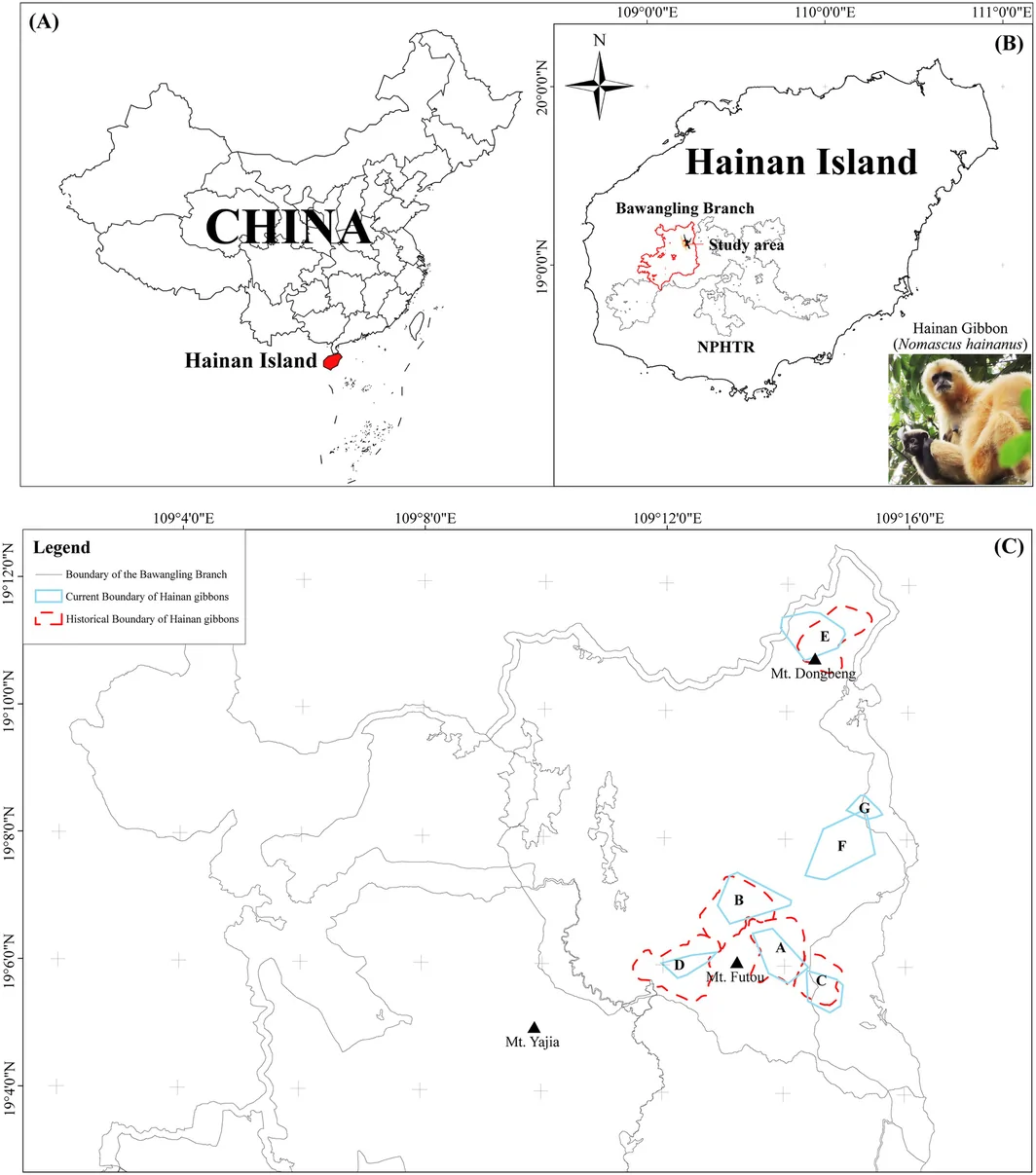
Study area in Bawangling branch of Hainan tropical rainforest national park (NPHTR), Hainan Island, China. (A) Location of Hainan Province within China. (B) Location of the Bawangling Branch and current study area within the National Park of Hainan Tropical Rainforest. (C) Spatial distribution of seven Hainan gibbon family groups (GA–GG), showing both historical (2000–2022, red dashed lines) and current (2023–2025, blue lines) group boundaries.

### 2.2 Research Scales

To investigate the ecological drivers of Hainan gibbon distribution across spatial scales, we conducted a two‐year monitoring study (March 2023–March 2025) combining landscape‐scale grid sampling with intensive intra‐group behavioral observations. Our study encompasses both population and group scales:

1. Population Scale: This level focuses on broad ecological dynamics influencing the entire Hainan gibbon population, including climate variability and landscape‐level habitat changes. We analyzed the relationship between the distribution of all seven social groups (Figure 2A) and 23 ecological variables (Table 1).
2. Group Scale: Among the seven groups, GA–GD primarily inhabit relatively mature forests with higher food plant diversity (Zhang et al. 2022; Lu et al. 2025), while GE–GG occupy secondary forests, including large areas regenerated from historical pine plantations. To represent these contrasting habitat conditions, we selected GC, which occupies relatively mature secondary forest with higher vegetation complexity, to represent GA–GD, and GE, which inhabits pine‐dominated secondary forest, to represent GE–GG (Liu, Zhang, Ameca, et al. 2025). We examined activity frequency—defined as the number of observed locations involving major behaviors (feeding, traveling, resting, and socializing)—in relation to ecological variables (Figure 2B). This scale captures how family groups adapt to microhabitats and ecological pressures, providing insight into behavioral adaptation, niche differentiation, and resource partitioning.

**FIGURE 2:**
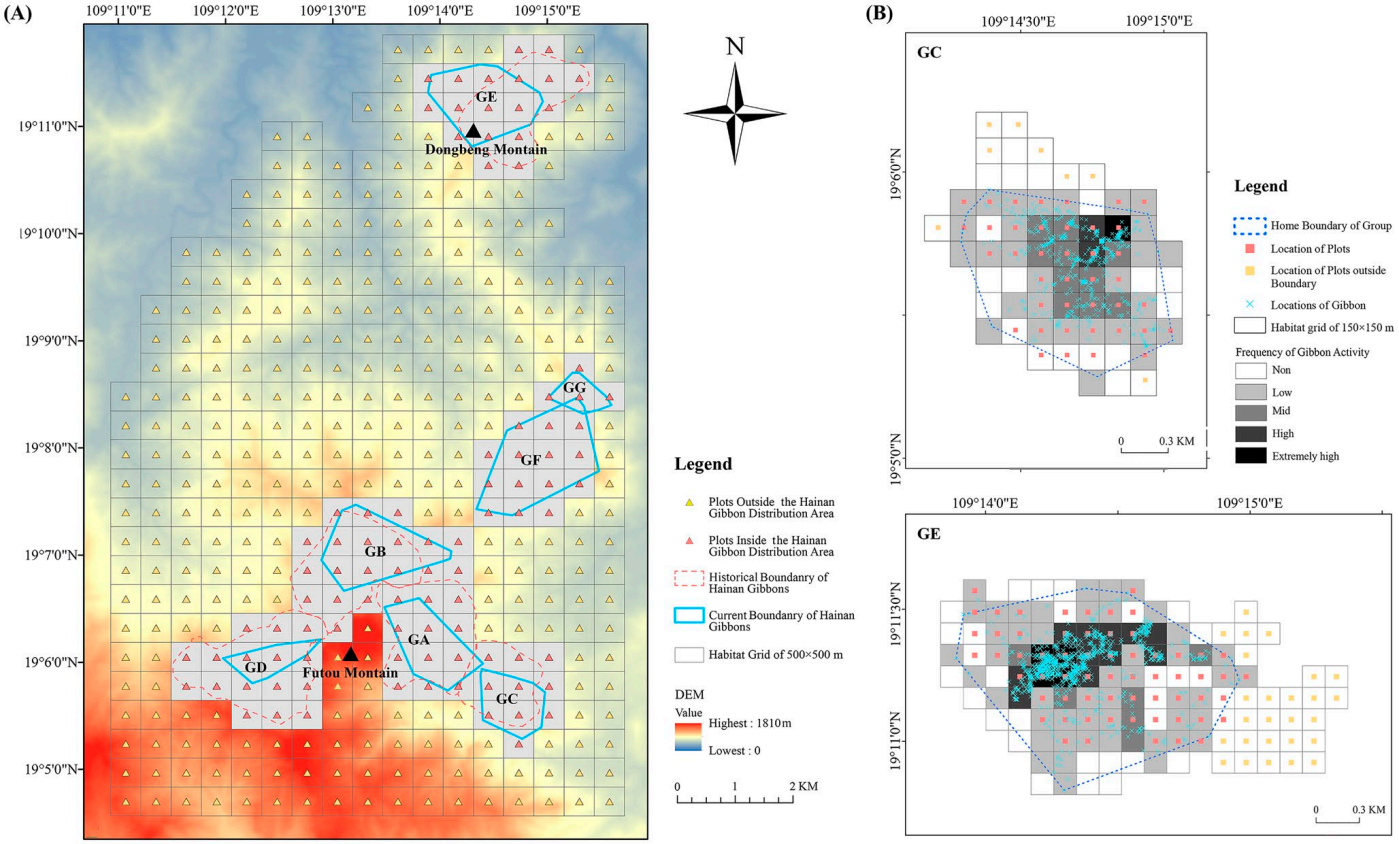
Spatial distribution patterns of Hainan gibbon. (A) Population scale: Distribution of the Hainan gibbon population (2000–2025) and the study plot grid layout (500 m × 500 m). (B) Group scale: Activity frequency distribution of the Hainan gibbon in groups GC and GE (2023–2025), and the corresponding plot grid layout (150 m × 150 m). Empty grid cells represent areas where no sampling plots were established and no gibbon activity locations were recorded during the study period.

**TABLE 1:** Selection and significance of ecological variables.

Categories	Ecological variables	Data acquisition method	Significance
Climate	Annual mean temperature	Climatic variables were obtained from the CHELSA v2.1 database (30 arc‐second resolution, ~1 km), which provides high‐resolution global climate surfaces representing long‐term average climatic conditions	Determines habitat climate conditions and seasonal resource availability
	Isothermality		
	Annual precipitation		
	Precipitation seasonality		
Land cover	Normalized Difference Vegetation Index (NDVI)	NDVI, NDWI, and land‐cover data (GLCC) were obtained from the USGS EarthExplorer platform (1 km spatial resolution). Forest type information was obtained from the Hainan Provincial Second‐Class Forest Inventory Dataset	Reflects vegetation productivity, water availability, land use patterns, and forest succession status in habitats
	Normalized Difference Water Index (NDWI)		
	Land Cover (LC; remote‐sensing derived)		
	Forest type (primary forest and secondary forest)		
Topography	Elevation	Elevation, slope, aspect, and slope position were measured directly within each plot using a Leica TPS1200+ total station and a Suunto PM‐5 inclinometer (see Section 2.5)	Influences microclimatic conditions, accessibility, and plant distribution affecting gibbon habitat use
	Slope		
	Aspect		
	Slope position		
Community structure	Diameter at breast height (DBH)	DBH, tree height (H), crown area, and crown height were measured in the field within each plot (see Section 2.5) Canopy density was estimated using hemispherical photography: five upward‐facing fisheye images were taken at the center and corners of each plot, processed in Adobe Photoshop to calculate the proportion of sky pixels (canopy openness), and averaged across the five images. Canopy density was then calculated as: Canopy Density = 1 − Canopy Openness	Reflects forest structural stability relevant to gibbon movement and shelter
	Tree height (H)		
	Crown area		Influences predator avoidance and microclimatic regulation
	Crown height		
	Canopy density		
Plant diversity	Community plant abundance (CP Abundance)	α‐diversity indices (including species richness, Shannon‐Wiener index, and Pielou's evenness) were calculated following the method of Ma (1994)	The diversity, complexity, and evenness of habitat plant communities
	Community plant richness (CP Richness)		
	Shannon‐Wiener index (Shannon)		
	Pielou index (Pielou)		
Food	Food plant abundance (FP Abundance)	Food plant diversity was determined based on two‐year direct feeding observations (2023–2025) combined with long‐term dietary monitoring records from the Bawangling division of the NPHTR and literatures (Zhang et al. 2022; Zhong et al. 2023)	Represents food resource availability
	Food plant richness (FP Richness)		

### 2.3 Delineation of Hainan Gibbons' Home Range

Based on location monitoring data of the seven Hainan gibbon groups (March 2023–March 2025), we used ArcGIS 10.8 with the Home Range Tool (HRT) v2.0 to delineate home range boundaries using the 95% Minimum Convex Polygon (MCP) method (Figure 2A, blue outlines). For groups with limited observation duration (GA, GB, and GD) and to better capture long‐term fidelity typical of territorial primates, we incorporated historical home range data. These data, compiled from nearly 20 years of monitoring by the Bawangling division of the NPHTR (2000–2022), were combined with digital topographic maps and satellite imagery to estimate pre‐2023 range extents for groups A–E (Figure 2A, red dashed lines; Zhang et al. 2022).

### 2.4 Activity Monitoring for GC and GE

From March 2023 to March 2025, we conducted monthly activity observations of the GC and GE gibbon groups. Each group was monitored by two trained observers: one tracked group activities and the other recorded location data. Prior to formal data collection, all observers completed calibration exercises to ensure inter‐observer reliability, achieving agreement rates above 85%, consistent with behavioral field standards (Altmann 1974; Martin and Bateson 2007). Monitoring occurred from the 1st to 15th of each month, starting around 5 AM at listening posts to detect initial calls and continuing until the gibbons entered their sleeping trees or the group lost visibility. We used instantaneous scan sampling every 5 min to record individual activities and associated locations (Chen et al. 2020). GC was monitored for 937 h over 183 days, yielding 17,818 records; GE was monitored for 987 h over 227 days, yielding 19,829 records.

### 2.5 Layout and Survey of Habitat Plots

Following the plot establishment standards of the Center for Tropical Forest Science (CTFS) at the Smithsonian Tropical Research Institute, we used the fishnet tool in ArcGIS 10.8 and a Leica TPS1200+ total station (Leica Geosystems, Heerbrugg, Switzerland) to set up monitoring plots in the study area.

With focus on the population level scale, we established 395 permanent plots, each measuring 20 m by 20 m and spaced 0.5 km apart, in and around the distribution area of the Hainan gibbon. Specifically, 93 plots were positioned within the core habitat of the gibbons, and 302 were located in adjacent areas, covering a total area of 15.8 ha (Figure 2A). Each plot was further divided into 16 quadrats (5 m × 5 m) to facilitate the mapping and systematic measurement of individual trees and improve the accuracy of vegetation structure assessments (Condit 1998; Yang et al. 2023). In each quadrat, every tree and liana with a diameter at breast height (DBH) of 1 cm or greater were identified, tagged, and positioned. DBH was measured using a diameter tape; tree height, tree crown height, and tree crown width were measured using a Nikon Forestry Pro laser rangefinder; and tree crown area was calculated based on perpendicular crown diameters. Topographic variables including elevation and aspect were recorded using a total station (Leica TPS1200+, Leica Geosystems, Heerbrugg, Switzerland), while the slope was measured using a Suunto PM‐5 inclinometer. Slope position (i.e., topographic position) was determined through field‐based geomorphological assessment following standard ecological classification (i.e., ridge, upper slope, middle slope, lower slope, valley; Han et al. 2024). Data collected per plot included species names, community structure variables (i.e., DBH, tree height, tree crown height, and tree crown width), and topographic variables (i.e., elevation, aspect, slope, and slope position; Figure 2A). Measurements from quadrats were aggregated to produce 20 m × 20 m plot‐level values, which served as the analysis unit for vegetation and topographic metrics. Each plot was located within a corresponding 500 × 500 m grid cell and was used to characterize local habitat conditions, which were then linked to environmental variables at the grid scale for population‐level analysis.

With focus on the group level, permanent plots, each measuring 50 m by 50 m, were established every 100 m using the total station in the territories of the GC and GE family groups and their surrounding areas, with 48 plots in the GC group's territory and 88 in the GE's group territory. Each of these larger plots was subdivided into 25 smaller quadrats of 10 m by 10 m to facilitate measurement. Given that the Hainan gibbon predominantly resides in large trees, this study focused on large trees, lianas, and shrubs with a DBH of 10 cm or greater as research subjects at this scale (Fan et al. 2011). Data collection followed the same protocol as used in the population‐level plots (Figure 2B).

### 2.6 Selection of Ecological Variables

To comprehensively investigate the ecological mechanisms underlying habitat use and activity patterns of the Hainan gibbon, we selected 23 ecological variables spanning six major categories: climate, land cover, topography, community structure, plant diversity, and food. These variables were chosen based on ecological theory and prior empirical studies demonstrating their relevance to primates' habitat preference, spatial distribution, and conservation planning for primates (Fan and Jiang 2008; Yang et al. 2022; Zhang et al. 2022, 2024; Zhong et al. 2023). Together, they represent key dimensions of habitat quality, structural complexity, and resource distribution at both local and landscape scales (see Table 1).

### 2.7 Data Analysis

#### 2.7.1 Normalization and Calculation of Ecological Variables

1. Due to the varied sources and characteristics of the ecological variables, continuous predictors were standardized using z‐score scaling, while categorical variables were coded as factors to meet the requirements of statistical analysis. To ensure consistency among predictors with different spatial resolutions, all environmental variables were resampled or aggregated to the 500 m × 500 m analysis grid used in population‐scale analyzes. Remote sensing variables (NDVI and NDWI) were standardized using min–max normalization as follows:

(1) $$\text{Value}=\left(\frac{\left({\mathrm{DN}}_{\mathrm{raw}}‐{\mathrm{DN}}_{\min}\right)}{\left({\mathrm{DN}}_{\max}‐{\mathrm{DN}}_{\min}\right)}\times \left({\text{scale}}_{\max}‐{\text{scale}}_{\min}\right)‐1\right)$$

*DN*_raw_ represents the raw numerical measurements, and *DN*_max_ and *DN*_min_ represent the maximum and minimum of the raw numerical measurements.

2 Categorical variables include: land cover (1 = Irrigated Cropland and Pasture; 2 = Cropland/Grassland Mosaic + Grassland; 3 = Evergreen Broadleaf Forest), slope aspect (1 = North; 2 = Northeast; 3 = East; 4 = Southeast; 5 = South; 6 = Southwest; 7 = West; 8 = Northwest), and slope position (1 = Lower slope; 2 = Mid slope; 3 = Upper slope)
3 Among plant species diversity indices, the abundance index represents the species abundance in a plot, the richness index (S) represents the number of species present in a plot, the Shannon‐Wiener index reflects the diversity and complexity of plants within each plot, and the Pielou index represents the evenness of species within the plot. All these indices were calculated based on the alpha diversity calculation methods (Ma 1994).

#### 2.7.2 Analysis of Spatial Distribution Patterns of the Hainan Gibbon

We used the ArcGIS Fishnet tool to establish two sampling grids: 500 m × 500 m grids for population‐scale analysis (Figure 2A) and 150 m × 150 m grids for group‐scale habitat assessment (Figure 2B). At the population scale, we generated heatmaps to visualize the distribution characteristics of the Hainan gibbon, highlighting the presence or absence of the species. At the group scale, we applied Point Pattern Analysis (PPA) to annual monitoring data to assess the activity intensity within each 150 m × 150 m grid cell. The resulting heatmaps revealed the activity patterns and differences among GC and GE groups. Activity frequencies were categorized for visualization using the natural breaks (Jenks) classification method (Jenks 1967) to be classified into “Extremely high”, “High”, “Mid”, “Low”, and “Non” (Zhang et al. 2024; Figure 2B). This method optimizes class separation by minimizing within‐group variance and maximizing between‐group variance, providing a clear representation of spatial intensity patterns.

#### 2.7.3 Analysis of Distribution Drivers for the Hainan Gibbon Across Scales

To identify the ecological drivers shaping Hainan gibbon distribution at different spatial scales, we first assessed pairwise correlations among all candidate predictors using Pearson correlation analysis. Variables with high collinearity (|r| > 0.9) were excluded to reduce the effects of multicollinearity on subsequent model fitting (Dormann et al. 2013). Correlation structures were visualized using the *corrplot* package in R (Figures S1.1–S1.3). To further evaluate multicollinearity among retained predictors, variance inflation factors (VIFs) were calculated. Variables with VIF ≥ 5 were excluded from further analyzes, ensuring that explanatory variables entering the candidate models were statistically independent. When all predictors satisfied this criterion, they were retained simultaneously for model construction.

1. Population‐scale analysis:

At the population scale, the response variable was Hainan gibbon occurrence, coded as presence (1) or absence (0) within each 500 m × 500 m grid. Explanatory variables included 23 ecological factors spanning climatic conditions, land cover, topography, vegetation structure, plant diversity, and food availability (Table 2). Because many predictors exhibited non‐normal distributions according to Shapiro–Wilk tests (*p* < 0.05; Table S2.1), and because nonlinear relationships between ecological variables and species occurrence were expected, we employed generalized additive models (GAMs; Hastie and Tibshirani 1986). GAMs allow flexible modeling of nonlinear effects by combining linear predictors with smooth functions and are well suited for ecological presence–absence data.

**TABLE 2:** Analysis of ecological variables driving the distribution of the Hainan gibbon population using a generalized additive model (GAM) and variance decomposition.

Linear terms						Variance explained
Ecological variables	Estimate	SE	*z* value	Pr(>|*z*|)	Sig.	*R* ^2^_var_ (%)
NDVI	1.31	0.48	2.73	< 0.01	**	6.50
LC_Cropland/Grassland Mosaic	−1.16	0.80	−1.44	0.15		1.31
LC_Cropland/Woodland Mosaic						
LC_Grassland						
LC_Savanna						
LC_Evergreen Broadleaf Forest	1.15	1.70	0.68	0.50		
Forest Type_Primary Forest	0.97	0.77	1.27	0.20		0.80
Annual Precipitation	31.60	5.75	5.49	< 0.001	***	14.53
Elevation	3.22	1.01	3.20	< 0.01	**	11.16
Aspect_Northeast	−0.59	1.00	−0.59	0.55		2.81
Aspect_East	−1.55	0.92	−1.68	0.09		
Aspect_Southeast	−0.88	1.07	−0.82	0.41		
Aspect_South	0.62	1.02	0.61	0.54		
Aspect_Southwest	−0.35	1.03	−0.34	0.73		
Aspect_West	2.56	1.20	2.14	0.03	*	
Aspect_Northwest	−2.35	1.14	−2.06	0.04	*	
DBH	−0.39	0.52	−0.76	0.45		3.93
H	0.32	0.61	0.53	0.60		2.47
Crown Area	−0.68	0.54	−1.27	0.20		0.58
Crown Height	0.75	0.64	1.16	0.24		0.96
CP Abundance	0.02	0.55	0.04	0.97		1.15
CP Richness	−0.96	1.51	−0.63	0.53		2.70
Shannon	2.00	3.15	0.64	0.53		3.28
Peilou	−2.51	2.22	−1.13	0.26		2.47
FP Abundance	0.80	0.44	1.80	0.07		1.33
FP Richness	0.84	0.46	1.81	0.07		5.09

Smooth terms						
Ecological variables	edf	Ref.df	*χ* ^2^	*p*	Sig.	
NDWI	2.41	2.91	7.00	0.07		1.58
Annual mean temperature	3.03	3.53	31.32	< 0.001	***	12.95
Isothermality	3.55	3.87	13.80	0.01	*	5.62
Precipitation seasonality	3.26	3.67	30.46	< 0.001	***	11.99
Slope	2.53	3.05	3.92	0.26		6.78

*Note:* GAM Model Parameters: Adj.*R* ^2^ = 0.73; Deviance Explained = 73.2%; Scale Estimate = 1; UBRE = 0.65. Displayed are the standardized regression coefficients, representing the average parameter estimates for each variable within a Generalized Additive Model (GAM) analyzing the spatial distribution of the Hainan Gibbon. Detailed alongside are the 95% confidence intervals and the proportionate influence of each factor, expressed as the percentage of the model's explained variance. The Adj.*R* ^2^ for the consolidated model delineates the goodness‐of‐fit, while the significance of individual predictors is marked with asterisks, where * indicates *p* < 0.05, ** denotes *p* < 0.01, and *** signifies *p* < 0.001. The linear predictors are quantified by their fixed coefficients, and the smooth terms are elucidated by their effective degrees of freedom (edf), showcasing the non‐linear dynamics within the model.

A binomial error distribution with a logit link function was used. Candidate models were compared using Akaike's Information Criterion (AIC), which provides an effective balance between model fit and complexity in ecological datasets with moderate sample sizes and multiple predictors. The model with the lowest AIC value was selected as the best‐supported model describing population‐level distribution patterns.

2 Group‐scale analysis:

At the group scale, analyzes focused on intra‐group activity frequency, measured as the number of activity records per 150 m × 150 m sampling unit. Because climatic and land‐cover variables showed limited variation at this fine spatial scale, explanatory variables were restricted to 15 local habitat factors, including topography, vegetation structure, plant community characteristics, and food resource availability.

After applying Pearson correlation analysis and VIF screening (VIF < 5), we used multiple linear regression models (LMs) to evaluate the effects of fine‐scale ecological variables on group‐level activity frequency. Residuals were inspected using QQ plots (Figures S2.2 and S2.3) and showed approximate normality, supporting the use of LMs. Model selection was conducted using AIC to identify the most parsimonious model retaining ecologically meaningful predictors. Regression coefficients were interpreted as the effect of each localized ecological variable on gibbon activity frequency at the group‐scale plots.

#### 2.7.4 Definition of Ecological Weighted Index and Predictive Model Calibration

To quantify the relative contributions of individual ecological variables to Hainan gibbon distribution and activity patterns across scales, we applied variance decomposition using the *GLMM.hp* package in R version 4.3.3 (Lai et al. 2022). This approach decomposes the adjusted coefficient of determination (adjusted *R* ^2^) into independent contributions from each predictor, providing an objective measure of variable importance. Based on these results, we constructed an ecological weighted index to integrate multiple ecological drivers into a single metric of habitat suitability. In ecological studies, weighting predictors by their explanatory power facilitates synthesis and improves predictive performance (Elith et al. 2008). Here, the percentage contribution of each variable derived from variance decomposition was used as its weight. The ecological weighted index (Index) was calculated as:

(2) $$\text{Index}=\sum \left(I_{\text{perci}}\times \text{scale}\left(X_{i}\right)\right)\%$$

where *I*_perci_ represents the percentage importance of each factor derived from variance decomposition, and scale (*X*_*i*_) represents the standardized value of each factor.

This index was used to evaluate habitat suitability and predict Hainan gibbon distribution across spatial scales. At the population scale, logistic regression was used to model the relationship between the ecological weighted index and species occurrence. At the group scale, because activity frequency data—measured as continuous counts—exhibited overdispersion (variance exceeding the mean), we applied negative binomial regression, which is more appropriate than Poisson regression for such count data (James et al. 2013). These models were used to fit predictive curves describing scale‐specific responses to habitat suitability. All statistical analyzes were conducted using R version 4.3.3 and ArcGIS 10.8.

## 3 Results

### 3.1 Spatial Patterns of the Hainan Gibbon Across Scales

Based on historical records combined with MCP analyzes, the current distribution of the Hainan gibbon population encompasses approximately 23.25 km^2^, corresponding to 93 population‐scale grid cells (Figure 2A). The cumulative distribution area of five family groups documented prior to 2023 was 11.58 km^2^, with individual home ranges ranging from 1.44 to 2.88 km^2^ (*n* = 5; Table S3.1). MCP analyzes of monitoring data collected between 2023 and 2025 indicated that core activity areas of the seven extant groups collectively covered 0.90 km^2^, with substantial variation among groups (*n* = 7; range = 0.41–2.42 km^2^), partly reflecting differences in observation intensity and habitat accessibility.

At the group scale, GC and GE exhibited contrasting spatial distribution patterns (Figure 2B). GC occupied 42 grid cells, including one classified as “extremely high” and two as “high” activity, with activity concentrated in a small number of core areas. In contrast, GE occupied 69 grid cells, including 4 “extremely high” and 11 “high” activity cells, distributed across multiple sections of the home range. Overall, GC displayed a more spatially concentrated distribution, whereas GE exhibited a broader and more spatially dispersed pattern.

### 3.2 Drivers of Hainan Gibbon Distribution Across Scales

#### 3.2.1 Drivers of Hainan Gibbon Distribution at the Population Scale

At the population scale, Hainan gibbon distribution was primarily influenced by landscape, climatic, and topographic variables, whereas plant community structure and species diversity contributed comparatively less to the fitted model (Table 2). Among land‐cover variables, NDVI was positively associated with gibbon occurrence (*p* < 0.01). Climatic variables, including annual precipitation (*p* < 0.001), annual mean temperature (*p* < 0.001), and precipitation seasonality (*p* < 0.001), showed strong associations with gibbon distribution, while isothermality was negatively correlated (*p* = 0.01). Elevation was also positively associated with gibbon occurrence (*p* < 0.01).

Variance decomposition indicated that climatic variables accounted for the largest proportion of explained variance, with annual precipitation (*R* ^2^_var_ = 0.15), annual mean temperature (*R* ^2^_var_ = 0.13), and precipitation seasonality (*R* ^2^_var_ = 0.12) showing the highest individual contributions (Table 2).

#### 3.2.2 Drivers of Hainan Gibbon Distribution at the Group Scale

Distinct differences in habitat associations were observed between the GC and GE family groups. In GC, activity frequency was positively associated with elevation (*β* = 0.51, *p* = 0.04) and food plant richness (*β* = 1.17, *p* < 0.01). Variance decomposition revealed that plant diversity variables collectively explained the largest proportion of variance (*R* ^2^_var_ = 0.60), with food plant richness contributing the most (*R* ^2^_var_ = 0.47), followed by tree height (*R* ^2^_var_ = 0.12), crown area (*R* ^2^_var_ = 0.10), and elevation (*R* ^2^_var_ = 0.09; Figure 3A; Table S4.1).

**FIGURE 3:**
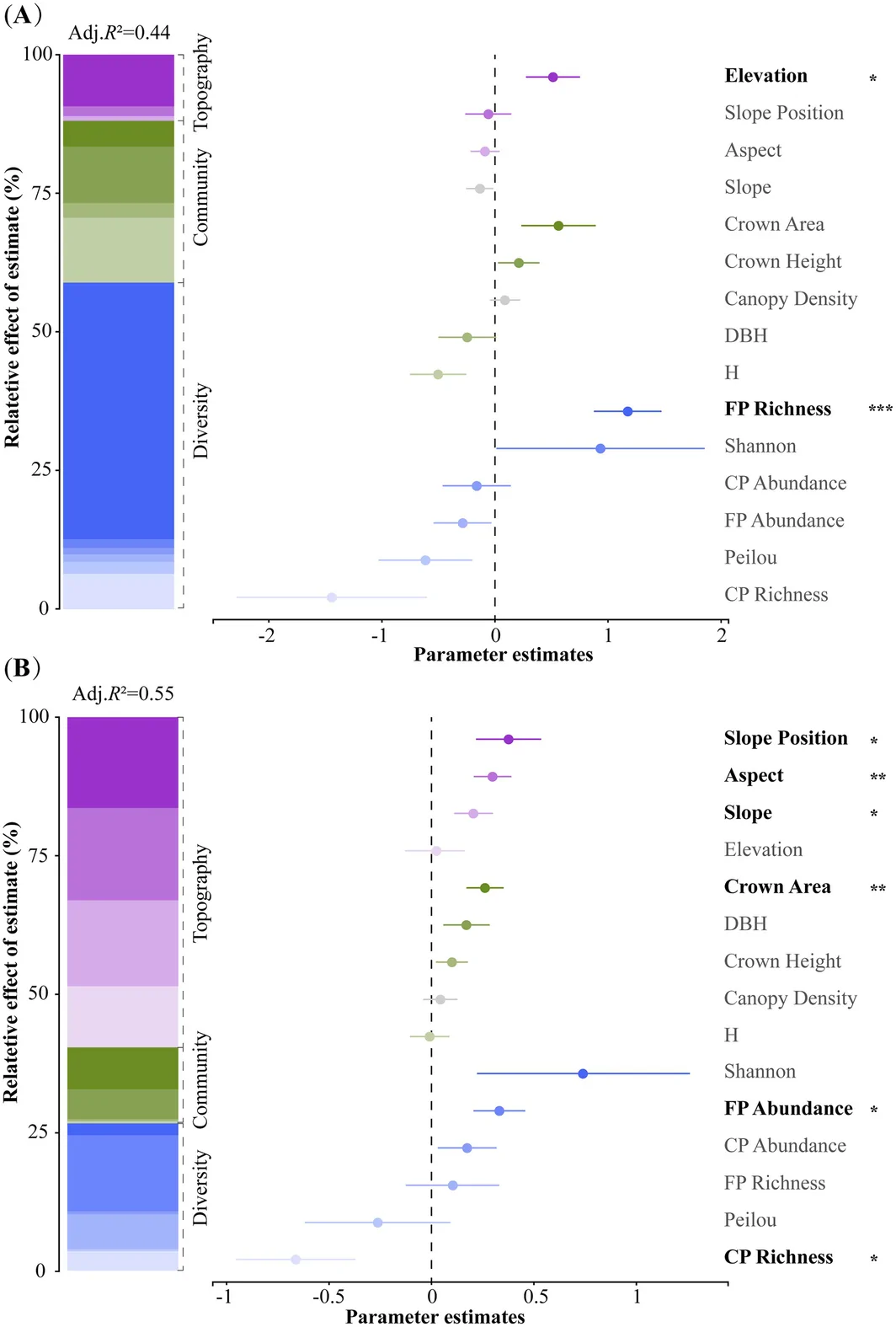
Effect of topography, plant community and plant diversity variables on Hainan gibbon activity frequency in groups GC (panel A) and GE (panel B). Average parameter estimates (standardized regression coefficients) of model predictors, associated 95% confidence intervals and relative importance of each factor are expressed as the percentage of explained variance. The adjusted (adj.) *R* ^2^ of the averaged model and the *p* value of each predictor are given as: *p < 0.05; **p < 0.01. Different colors represent different driving factors and their categories; gray indicates that the factor contributes no or negative variance to the total model variance. Detailed model coefficients are provided in Table S4.1. CP Abundance, community plant abundance; CP Richness, community plant richness; DBH, diameter at breast height; FP Abundance, food plant abundance; FP Richness, food plant richness; H, tree height; Pielou, Pielou index; Shannon, Shannon.wiener index.

In GE, activity frequency was positively associated with slope (*β* = 0.21, *p* = 0.03), slope position (*β* = 0.38, *p* = 0.02), food plant abundance (*β* = 0.33, *p* = 0.01), slope aspect (*β* = 0.30, *p* < 0.01), and crown area (*β* = 0.26, *p* < 0.01). Variance decomposition showed that topographic variables explained the largest proportion of variance (*R* ^2^_var_ = 0.60). Among individual predictors, slope aspect had the highest explanatory power (*R* ^2^_var_ = 0.17), followed by slope position (*R* ^2^_var_ = 0.16), slope (*R* ^2^_var_ = 0.16), and food plant abundance (*R* ^2^_var_ = 0.14; Figure 3B; Table S4.1).

### 3.3 Predictive Modeling of Hainan Gibbon Activity Using a Weighted Ecological Index Across Spatial Scales

The weighted ecological index revealed clear differences in habitat suitability patterns across spatial scales. At the population scale, logistic regression (*AIC* = 250.61) showed a nonlinear increase in gibbon occurrence probability with increasing index values (Figure 4A). At the group scale, activity frequency increased more steeply with the ecological index in GE than in GC (Figure 4B). Negative binomial regression indicated a better model fit for GC (AIC = 279.80) than for GE (AIC = 392.49), reflecting differences in habitat constraints between groups.

**FIGURE 4:**
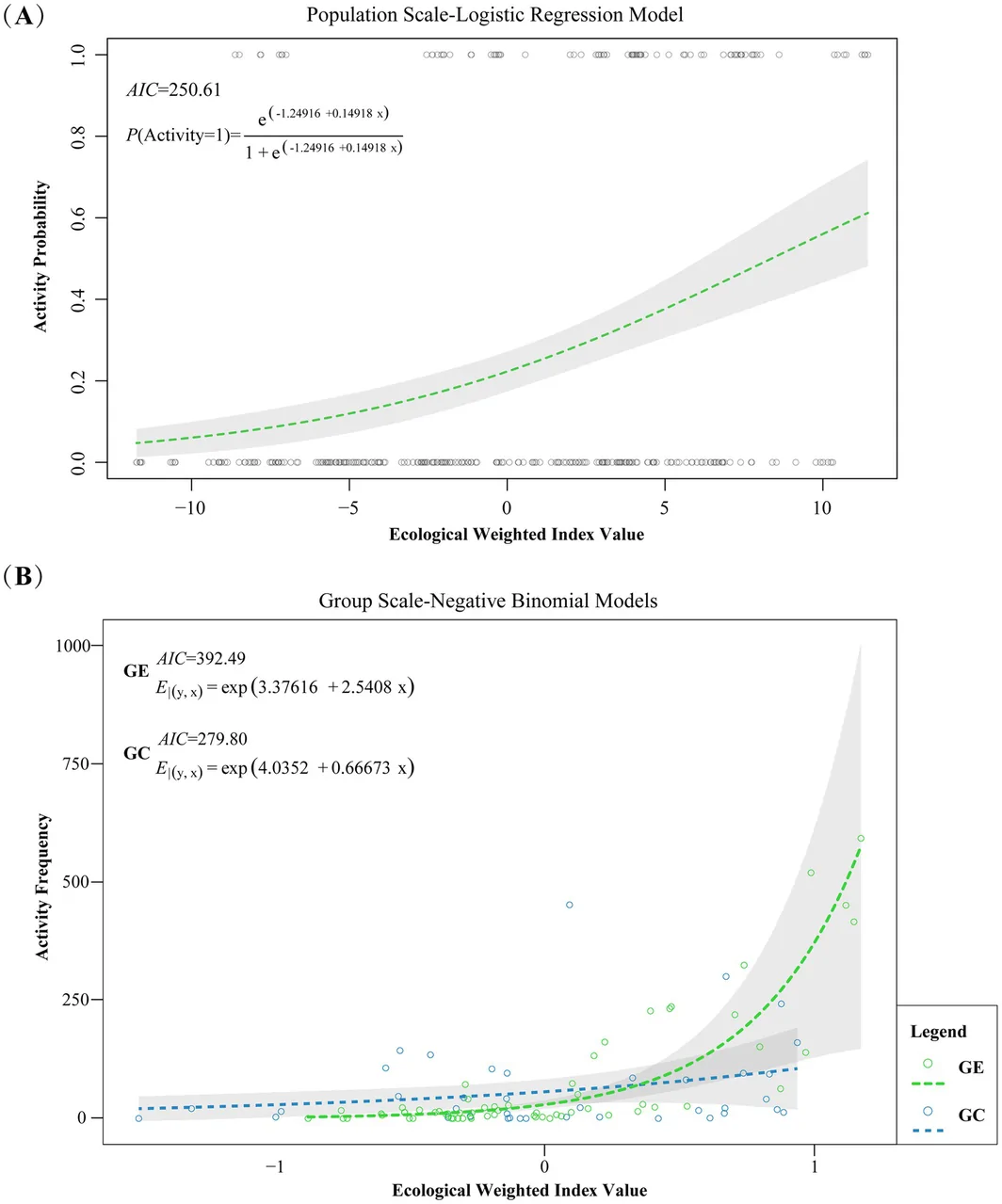
Predictive modeling of Hainan gibbon activity using a weighted ecological index across spatial scales: Population scale (panel A) and group scale (panel B). (A) Logistic regression model predicting activity probability of the Hainan gibbon as a function of the Ecological Weighted Index at the population scale. Activity probability increases with higher index values, indicating a positive association (intercept = −1.25, *β* = 0.15). Shaded area indicates 95% confidence intervals. Based on 395 population‐level sampling plots. (B) Negative binomial models predicting activity frequency as a function of the Ecological Weighted Index within groups GC and GE. In GE, activity frequency increases sharply (intercept = 3.38, *β* = 2.54), while in GC, activity increases more gradually (intercept = 4.04, *β* = 0.67). Model type, AIC values, regression formulas, and legend details are presented in the panels.

## 4 Discussion

### 4.1 Scale‐Dependent Ecological Preferences and Conservation Management

Focusing on the Critically Endangered Hainan gibbon, our study highlights how species distribution is shaped by both scale‐dependent and habitat‐specific ecological characteristics, underscoring the need for a multiscale conservation strategy that integrates fine‐scale habitat restoration with broader landscape connectivity planning.

At the population scale, we found that macro‐environmental variables—including NDVI (a proxy for vegetation productivity), annual precipitation, elevation, annual mean temperature, and precipitation seasonality—were significant determinants of gibbon distribution, yet these factors have often been underrepresented in previous studies. These variables govern key ecological processes that influence habitat carrying capacity and affect survival through bottom‐up mechanisms: for example, annual and seasonal precipitation shape phenology and the temporal availability of key food resources such as young leaves and fruits (Du et al. 2020); elevation and temperature gradients constrain energy budgets (Campos et al. 2017); and NDVI likely captures seasonal variation in vegetation greenness, which may reflect food availability and influence foraging behavior (Li et al. 2024). However, relying solely on NDVI may neglect critical vertical habitat structures essential for brachiation and foraging efficiency. Incorporating structural data from complementary sources such as LiDAR may improve the evaluation of habitat suitability. These findings align with Yang et al. (2022), who emphasized the role of climatic and vegetation factors in shaping large‐scale gibbon habitat patterns, and complement Zhang et al. (2023), who identified anthropogenic disturbances—such as habitat conversion and forest cover loss—as major drivers of Hainan gibbon distribution using species distribution models (SDMs).

At the group scale, finer‐scale habitat characteristics—including topography, crown area, and food plant diversity—emerged as key factors influencing gibbon activity (Figure 3). These variables are closely aligned with the ecological needs of individual gibbons, underscoring the critical role of microhabitat conditions in shaping group‐level behavior and survival strategies (Chapman et al. 2018; Ning et al. 2019). Such scale‐dependent habitat preferences are consistent with findings in other gibbon species. For instance, Nguyen et al. (2020) found that forest type strongly influenced the distribution of the southern white‐cheeked gibbon ( *Nomascus siki* ) in Vietnam's Truong Son Key Biodiversity Area (KBA). Similarly, Fan and Jiang (2008) reported that food resource distribution and topography determined the home range of the black‐crowned gibbon ( *Nomascus concolor* ). These parallels reinforce the need for conservation strategies tailored to different ecological scales. At the population level, expanding suitable habitat to maintain connectivity among vegetation and climate zones is essential for gene flow and long‐term population viability. At the group level, conservation actions should prioritize improving habitat quality by enhancing food plant diversity and restoring canopy complexity.

### 4.2 Habitat‐Specific Ecological Preferences of Species and Conservation Management

At the group scale, GC and GE exhibited distinct habitat selection preferences: GC favored areas characterized by higher elevation and greater food plant richness, whereas GE preferred sites with steeper slopes, higher slope positions, broader crown areas, and more abundant food plant resources. These differences likely reflect the contrasting habitat conditions occupied by the two groups. GC inhabits relatively mature forests with higher food plant diversity (Zhang et al. 2022), whereas GE occupies secondary forest regenerated from former pine plantations (Chan et al. 2020; Liu et al. 2023). As a result, gibbons in these differing habitats may exhibit divergent activity patterns to optimize survival.

Such patterns are consistent with findings from other primate species. For example, Guan et al. (2018) investigated gibbons in cold, seasonal forests of northern China—including *Nomascus concolor* , *Nomascus nasutus* , and *Hoolock tianxing* —and found that, compared to their tropical relatives, these gibbons coped with harsher environments and food scarcity by expanding their home ranges and increasing leaf consumption. Similarly, Ni et al. (2014) compared two *Nomascus concolor* groups in habitats of differing quality and found that the group in higher‐quality habitat consumed more non‐fig fruits, while the group in degraded areas relied more on young leaves to compensate for reduced fruit availability. These findings, in line with our results, highlight the role of habitat quality in shaping gibbon behavioral strategies. Accordingly, conservation planning must account for the specific ecological conditions of different groups to implement targeted and effective management interventions.

Behavioral patterns observed in the GC and GE groups further suggest different ecological mechanisms underlying habitat use. Optimal foraging theory predicts that animals maximize energy intake by selecting patches with higher resource availability (Chapman et al. 2024). In GC, activity patterns were most strongly associated with food plant richness and other plant diversity variables, suggesting that individuals may preferentially exploit areas offering a wider variety of food resources. Such patterns are broadly consistent with predictions of optimal foraging theory, where animals concentrate their activities in resource‐rich patches to maximize energy intake. In contrast, GE activity was more strongly associated with topographic features and canopy structure. These habitat characteristics can influence canopy connectivity and locomotion efficiency for arboreal primates (José‐Domínguez et al. 2015), while also potentially providing refuge from predators (Fan and Jiang 2008; Brividoro et al. 2019). Under such conditions, animals may adjust their movement patterns to reduce energetic costs, a pattern consistent with energy conservation strategies (Bach et al. 2017; Yang et al. 2026).

Together, these findings underscore the ecological flexibility of Hainan gibbons in response to habitat heterogeneity. Conservation strategies should adopt a habitat‐specific approach: for the GC group, priority should be placed on protecting areas with high food plant diversity, while for the GE group, management efforts should focus on maintaining structurally complex forest with rugged topography and enhancing food plant resources to support group persistence and growth. Such tailored management will be essential for improving habitat quality and securing the long‐term viability of this critically endangered species.

### 4.3 Lessons for Species Conservation Management in Fragmented Landscapes

Building on the scale‐dependent patterns identified above, our study provides a spatially explicit framework for multi‐scale conservation planning by revealing how scale‐dependent ecological factors shape the distribution of the Critically Endangered Hainan gibbon.

At the population scale, the probability of gibbon occupation increased exponentially with overall habitat suitability, underscoring the importance of broad‐scale ecological filters—such as macro‐climatic stability and vegetation cover—in shaping population‐level distributions. Areas characterized by moderate temperature variation, sufficient precipitation, and dense vegetation may represent priority zones for species range expansion. We recommend these areas be targeted for landscape‐level habitat protection and potential reintroduction, thereby supporting broader recovery efforts. These findings reinforce and extend earlier conservation modeling efforts (Fan et al. 2013). At the group scale, the contrasting responses of the GC and GE groups highlight the necessity of context‐specific interventions. In GE habitats, activity frequency exhibited a steep response to the ecological index, suggesting high responsiveness to habitat improvement. This pattern indicates that GE activity patterns are strongly influenced by local habitat conditions. Because some of the key predictors identified in the models—such as food plant abundance and canopy structure—are potentially manageable through habitat restoration, targeted management actions may improve habitat suitability in this area. Such measures may include enrichment planting of key food tree species (e.g., large‐fruiting figs; Zhang et al. 2022) and enhancing canopy connectivity to facilitate movement. In contrast, the relatively mature habitat of group GC displayed a more gradual, saturating response to the ecological index, indicating diminishing marginal returns from further habitat enrichment. For GC, conservation strategies should prioritize maintaining existing habitat stability rather than additional habitat enrichment, strengthening surveillance to prevent poaching, and minimizing ecological competition with sympatric species such as macaques (Cai et al. 2024). Similar patterns have been observed in other primates, where the retention of high‐quality feeding trees has proven vital to maintaining population persistence (Milton et al. 2019). Taken together, the population‐ and group‐scale analyzes provide complementary insights for conservation planning. Population‐scale patterns help identify priority areas for landscape‐level habitat protection and potential range expansion, whereas group‐scale results reveal habitat attributes influencing local activity patterns and management needs within occupied areas. These cross‐scale insights support evidence‐based habitat zoning and targeted conservation actions in fragmented landscapes.

## 5 Conclusion

By integrating two‐year intensive monitoring with ~20 years historical range information, we identified distinct ecological drivers shaping Hainan gibbon distribution across scales. Macro‐environmental variables (climate, NDVI, elevation) defined population‐level distribution, while topography and food plant composition governed local group activity. We developed a scale‐explicit habitat weighting approach that quantifies habitat suitability by integrating variable importance across nested scales. This dual‐scale approach revealed that population persistence depends on expanding climatically suitable habitats, while group viability requires targeted restoration of canopy and food resources. Our findings highlight the importance of integrating spatial scaling theory into conservation planning, providing a transferable model for species with limited distributions facing rapid environmental change.

## Author Contributions

**Shuai Liu:** conceptualization (equal), data curation (equal), funding acquisition (equal), investigation (equal), methodology (equal), validation (equal), writing – original draft (equal), writing – review and editing (equal). **Dexu Zhang:** data curation (equal), investigation (equal). **Chenglin Cai:** data curation (equal), investigation (equal). **Yidong Zhou:** data curation (equal), investigation (equal). **Kening Lu:** data curation (equal), investigation (equal). **Yuan Chen:** formal analysis (equal), supervision (equal), writing – review and editing (equal). **Wenxing Long:** conceptualization (equal), project administration (equal), supervision (equal), writing – review and editing (equal). **Eric I. Ameca:** supervision (equal), writing – review and editing (equal).

## Funding

This work was supported by the National Natural Science Foundation of China (U22A20503), the Hainan Provincial Science and Technology Talent Innovation Project (KJRC2023C13), Project of the Hainan University Collaborative Innovation Center (XTCX2022STC24), and the Hainan Provincial Graduate Innovation Research Project (Qhyb2022‐31).

## Ethics Statement

All fieldwork and behavioral observations involving Hainan gibbons were conducted in strict accordance with the Wildlife Protection Law of the People's Republic of China and relevant research ethics guidelines. Research permits and field access were approved by the Bawangling Branch of the Hainan Tropical Rainforest National Park Administration. No animals were captured, handled, or experimentally manipulated during the study.

## Conflicts of Interest

The authors declare no conflicts of interest.

## Supporting information


**Figure S1.1:** Pearson analysis of habitat variables in habitat at population scale. Variables with correlation coefficients greater than 0.9 were excluded to prevent the impact of spatial autocorrelation on regression results. All variables were retained. Abbreviations: Bio12 (Annual precipitation); ELE (Elevation); Shannon (Shannon. wiener index); CP Richness (Community plant richness); FP Richness (Food plant richness); Peilou (Peilou index); NDVI (Normalized Difference Vegetation Index); DBH (Diameter at breast height); Bio3 (Isothermality); NDWI (Normalized Difference Water Index); H (Tree height); FP Abundance (Food plant abundance); CP Abundance (Community plant abundance); GLCC (Global Land Cover Characterization); Bio15 (Precipitation seasonality); Bio1 (Annual mean temperature).
**Figure S1.2:** Pearson analysis of habitat variables in habitat within GC group. Variables with correlation coefficients greater than 0.9 were excluded to prevent the impact of spatial autocorrelation on regression results. Simpson index was removed and Shannon. wiener index was remained. Abbreviations are same as Figure S1.
**Figure S1.3:** Pearson analysis of habitat variables in habitat within GE group. Variables with correlation coefficients greater than 0.9 were excluded to prevent the impact of spatial autocorrelation on regression results. Simpson index was removed and Shannon.wiener index and Peilou index were remained. All variables were retained. Abbreviations are same as Figure S1.
**Table S2.1:** Shapiro–Wilk test for normality of habitat variables at population scale.
**Table S3.1:** Historical and current home range areas of Hainan gibbon family groups.
**Table S4.1:** Model coefficients and variance contributions of predictors for group‐scale drivers of Hainan gibbon activity frequency (GC and GE), corresponding to Figure 3A,B.

## Data Availability

All data and codes of this research are available at https://doi.org/10.6084/m9.figshare.32676963.
