# Supplementary material for: Multi‐Scale Habitat Drivers of Hainan Gibbon Distribution: Integrating Macro‐Environmental and Fine‐Scale Ecological Factors for Targeted Conservation of Threatened Species
Source: Ecol Evol. 2026 Oct 26;16(10):e74519. doi: 10.1002/ece3.74519 (PMC13710805; doi:10.1002/ece3.74519)
Supplement: Supplementary file 1 [file ECE3-16-e74519-s001.docx]

**Supplementary Information**

Multi-scale habitat drivers of Hainan gibbon distribution: Integrating macro-environmental and fine-scale ecological factors for targeted conservation of threatened species

This file contains detailed results on:

S1: Pearson correlation analysis of multi-scale habitat variables

S2: Normality test of multi-scale habitat variables

S3: Home range areas of Hainan gibbon family groups

S4: Model results for group-scale analyses

**S1. Pearson correlation analysis of multi-scale habitat variables**

**Fig. S1.1** **Pearson analysis of habitat variables in habitat at population scale.** Variables with correlation coefficients greater than 0.9 were excluded to prevent the impact of spatial autocorrelation on regression results. All variables were retained. Abbreviations: Bio12 (Annual precipitation); ELE (Elevation); Shannon (Shannon.wiener index); CP Richness (Community plant richness); FP Richness (Food plant richness); Peilou (Peilou index); NDVI (Normalized Difference Vegetation Index); DBH (Diameter at breast height); Bio3 (Isothermality); NDWI (Normalized Difference Water Index); H (Tree height); FP Abundance (Food plant abundance); CP Abundance (Community plant abundance); GLCC (Global Land Cover Characterization); Bio15 (Precipitation seasonality); Bio1 (Annual mean temperature).

**Fig. S1.2** **Pearson analysis of habitat variables in habitat within GC group.** Variables with correlation coefficients greater than 0.9 were excluded to prevent the impact of spatial autocorrelation on regression results. Simpson index was removed and Shannon.wiener index was remained. Abbreviations are same as Fig. S1.

**Fig. S1.3** **Pearson analysis of habitat variables in habitat within GE group.** Variables with correlation coefficients greater than 0.9 were excluded to prevent the impact of spatial autocorrelation on regression results. Simpson index was removed and Shannon.wiener index and Peilou index were remained. All variables were retained. Abbreviations are same as Fig. S1.

**S2. Normality test of multi-scale habitat variables**

**Table S2.1** Shapiro-Wilk test for normality of habitat variables at population scale

| **Variable** | **W** | **p_value** |
| --- | --- | --- |
| NDVI | 0.93 | ＜0.01 |
| NDWI | 0.88 | ＜0.01 |
| GLCC | 0.71 | ＜0.01 |
| Forest Type | 0.60 | ＜0.01 |
| Bio1 | 0.94 | ＜0.01 |
| Bio3 | 0.97 | ＜0.01 |
| Bio12 | 0.96 | ＜0.01 |
| Bio15 | 0.95 | ＜0.01 |
| ELE | 0.95 | ＜0.01 |
| Aspect | 0.90 | ＜0.01 |
| Slope | 0.99 | 0.03 |
| DBH | 0.89 | ＜0.01 |
| H | 0.99 | 0.04 |
| Crow Area | 0.98 | ＜0.01 |
| Crow Height | 0.99 | 0.12 |
| CP Abundance | 0.98 | ＜0.01 |
| CP Richness | 0.96 | ＜0.01 |
| Shannon | 0.99 | 0.11 |
| Peilou | 0.94 | ＜0.01 |
| FP Abundance | 0.93 | ＜0.01 |
| FP Richness | 0.92 | ＜0.01 |

*Notes.* The Shapiro-Wilk test was employed to evaluate the normality of the data, with the test statistic (W) ranging from 0 to 1. Higher W values indicate a closer approximation to a normal distribution. The associated p-value (p) determines whether the null hypothesis of normality can be rejected. If *p* > 0.05, the null hypothesis is not rejected, suggesting that the data conform to a normal distribution. Conversely, if *p* ≤ 0.05 , the null hypothesis is rejected, indicating a significant deviation from normality.

**Fig. S2.2 Q-Q plot for normality test of residuals in group GC.** Most points align well with the reference line, indicating approximate normality.

**Fig. S2.3 Q-Q plot for normality test of residuals in group GE.** Most points align well with the reference line, indicating approximate normality.

**S3. Home range areas of Hainan gibbon family groups**

**Table S3.1** Historical and current home range areas of Hainan gibbon family groups.

| **Group** | **Historical home range (km²)** | **Period** | **Current home range (km²)** | **Period** | **Data source** |
| --- | --- | --- | --- | --- | --- |
| GA | 2.26 | 2000–2022 | 1.31 | 2023–2025 | Long-term monitoring records + HRT v2.0 |
| GB | 2.88 | 2000–2022 | 1.83 | 2023–2025 | Long-term monitoring records + HRT v2.0 |
| GC | 1.44 | 2000–2022 | 0.94 | 2023–2025 | Long-term monitoring records + HRT v2.0 |
| GD | 2.52 | 2000–2022 | 0.52 | 2023–2025 | Long-term monitoring records + HRT v2.0 |
| GE | 2.48 | 2000–2022 | 1.73 | 2023–2025 | Long-term monitoring records + HRT v2.0 |
| GF | - | - | 2.42 | 2023–2025 | HRT v2.0 only |
| GG | - | - | 0.41 | 2023–2025 | HRT v2.0 only |

*Notes*. Historical home ranges were derived from long-term monitoring records (2000–2022), whereas current home ranges were estimated using MCP (95%) based on 2023–2025 location data.

**S4. Model results for group-scale analyses**

**Table S4.1** Model coefficients and variance contributions of predictors for group-scale drivers of Hainan gibbon activity frequency (GC and GE), corresponding to Figure 3A–B.

| **Group** | **Predictor** | **Estimate** | **Std. Error** | **t value** | **P value** | **Variance (%)** |
| --- | --- | --- | --- | --- | --- | --- |
| GC | Slope | -0.14 | 0.12 | -1.11 | 0.28 | 1.92 |
|  | Elevation | 0.51 | 0.24 | 2.15 | 0.04* | 7.31 |
|  | Aspect | -0.09 | 0.13 | -0.67 | 0.51 | 1.24 |
|  | Slope position | -0.06 | 0.20 | -0.29 | 0.77 | 0.85 |
|  | DBH | -0.25 | 0.26 | -0.96 | 0.34 | 3.49 |
|  | Tree height | -0.50 | 0.25 | -2.02 | 0.05 | 7.15 |
|  | Crown area | 0.56 | 0.33 | 1.71 | 0.10 | 8.01 |
|  | Crown height | 0.21 | 0.18 | 1.14 | 0.26 | 2.99 |
|  | Canopy density | 0.09 | 0.13 | 0.67 | 0.51 | 1.27 |
|  | Shannon | 0.93 | 0.94 | 1.00 | 0.33 | 13.29 |
|  | Pielou | -0.62 | 0.42 | -1.48 | 0.15 | 8.76 |
|  | CP abundance | -0.16 | 0.30 | -0.54 | 0.60 | 2.30 |
|  | CP richness | -1.45 | 0.84 | -1.71 | 0.10 | 20.58 |
|  | FP abundance | -0.29 | 0.26 | -1.13 | 0.27 | 4.10 |
|  | FP richness | 1.17 | 0.30 | 3.93 | <0.001*** | 16.73 |
| GE | Slope | 0.21 | 0.10 | 2.17 | 0.03* | 5.47 |
|  | Elevation | 0.02 | 0.15 | 0.11 | 0.91 | 0.46 |
|  | Aspect | 0.30 | 0.09 | 3.26 | <0.01** | 7.94 |
|  | Slope position | 0.38 | 0.16 | 2.37 | 0.02* | 10.01 |
|  | DBH | 0.17 | 0.11 | 1.51 | 0.14 | 4.55 |
|  | Tree height | -0.01 | 0.10 | -0.09 | 0.93 | 0.23 |
|  | Crown area | 0.26 | 0.09 | 2.88 | <0.01** | 6.96 |
|  | Crown height | 0.10 | 0.08 | 1.28 | 0.21 | 2.67 |
|  | Canopy density | 0.05 | 0.08 | 0.55 | 0.58 | 1.19 |
|  | Shannon | 0.74 | 0.52 | 1.42 | 0.16 | 19.68 |
|  | Pielou | -0.26 | 0.36 | -0.74 | 0.46 | 7.00 |
|  | CP abundance | 0.17 | 0.14 | 1.21 | 0.23 | 4.64 |
|  | CP richness | -0.66 | 0.29 | -2.28 | 0.03* | 17.63 |
|  | FP abundance | 0.33 | 0.13 | 2.62 | 0.01* | 8.82 |
|  | FP richness | 0.10 | 0.23 | 0.45 | 0.65 | 2.75 |

*Notes*. Variance (%) represents the relative contribution of each predictor derived from variance decomposition analyses shown in Figure 3.
